# Influence of InP coupling cavity on Fano resonance of sub wavelength MIM waveguide

**DOI:** 10.1038/s41598-021-90773-8

**Published:** 2021-06-02

**Authors:** Shihao Ban, Huangqing Liu, Shifang Xiao, Jingjing Mao, Jie Luo

**Affiliations:** 1 School of Physics and Electronics, Hunan University, Changsha, 410082 People’s Republic of China; 2 Huaihua Normal College, Huaihua, 418008 People’s Republic of China; 3 School of Material Science and Engineering, Central South University of Forestry and Technology, Changsha, 410004 Hunan People’s Republic of China

**Keywords:** Optics and photonics, Physics

## Abstract

In this paper, influence of InP coupling cavity on Fano resonance of sub wavelength MIM waveguide was studied by FDTD. It was observed that the resonant wavelengths of mode m_j_ (j = 1, 2, 3) were closely related with the height *H*_2_ of InP coupling cavity. In addition, before and after the addition of air cavity, the relative farfield intensities *I* was a function of height *H*_2_. Therefore, InP as discrete state could be used as the filling dielectrics of Fano resonance in the MIM waveguide.

## Introduction

Due to its attractive features and extensive applications, the metal–insulator–metal (MIM) waveguide^1–5^ were very popular with researchers. During the process of light propagation in the nano-photonic circuits, MIM waveguides could reduce energy loss. The MIM waveguide with coupling cavity could change its filtering performance^6–9^, such as Fano resonance. The most common structures with the filters were MIM waveguides with a rectangular cavity^10–14^. Silicon was used as filter dielectrics in the coupling cavity^13,14^. Due to relatively large dielectric constant and high-order resonance about 1700 nm in the near-infrared region, silicon was usually regarded as discrete state of Fano resonance^13–18^. In the study, obvious Fano resonance could be observed when InP was chose as the dielectrics in the coupling cavity of MIM waveguide. Namely, InP could also be used as discrete state of Fano resonance. In this paper, InP^19–25^ would be used as the filling dielectrics in the MIM waveguide cavity and the influence of InP coupling cavity on Fano resonance of sub wavelength MIM waveguide (Ag-Air-Ag) was explored on the basis of the literatures^22,25,26^.

## Results

**Figure 1:**
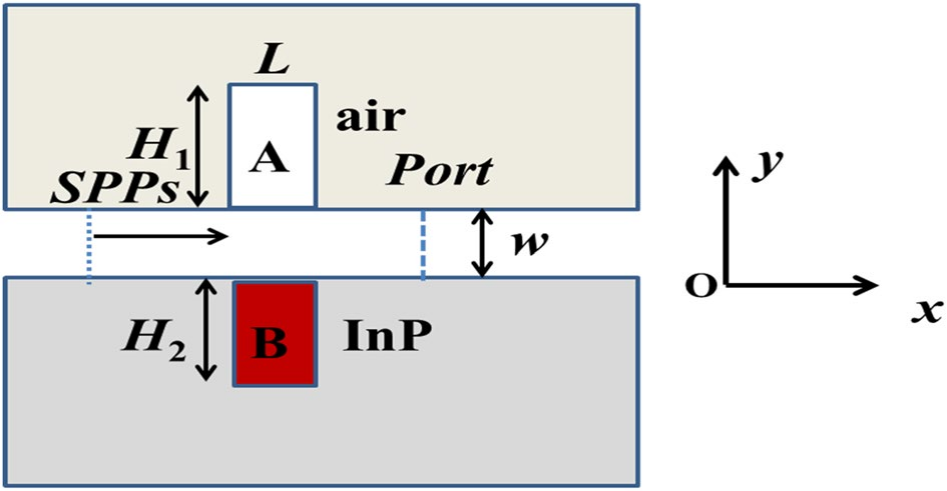
Research scheme and design schematic of 2D simulation. The capital letters A and B denote air and InP coupled cavities, respectively. The structural parameters were *L* = *w* = 50 nm.

The sketch of the waveguide structure designed was shown in Fig. 1. In the figure, the distance *w* of the main waveguide cavity with the transparent dielectrics (n = 1.0) was set as 50 nm which was far less than the wavelength *λ* of incident wave so that SPPs propagation mode could be excited in MIM waveguide structure because the number of SPP modes was closely related to the distance along the wave propagation direction^27^. In addition, the width ***L*** of the coupled cavities was also set as 50 nm. Our simulation results indicated that Fano resonance was independent of the horizontal distance between the two cavities and dependent on the height ***H***_1_ (***H***_**2**_). Additionally, the relative far-field intensity *I* was defined as the area under the far-field curve and was proportional to the height *h* of the curve in this paper.

**Figure 2:**
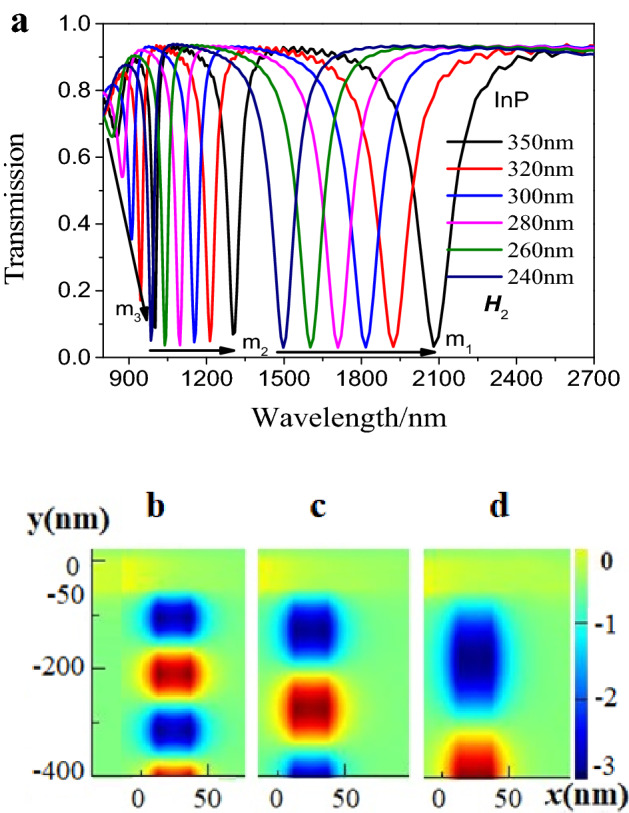
(**a**) The relation between Fano resonance and the height *H*_2_. (**b**–**d**) Magnetic field distribution of different modes at different resonance wavelengths 998 nm, 1302 nm and 2079 nm, respectively. Red and blue represent two different vibrations. The structural parameter was *H*_2_ = 350 nm.

Figure 2a showed the transmission spectra under different InP structures without air cavity **A**. It could be observed that the resonance peaks of mode m_j_ (j = 1, 2, 3) redshifted with the increase of *H*_2_ from 240 to 350 nm. And that the resonant wavelengths were a function of the height *H*_2_. Take *H*_2_ = 350 nm as an example, it could be observed three resonance valleys at about 998 nm, 1302 nm and 2079 nm, respectively. The resonance valleys at 1302 nm and 2079 nm were considered to be first and second order resonance modes, shown in Fig. 2c,d. However, the resonance valley at 998 nm was regarded as third-order resonance (seen in Fig. 2a,b), which was independent of the height ***H***_1_ and closely related to the height* H*_2_ according to our simulations. In other words, the resonance mode in Fig. 2a could directly be confirmed by the distribution of magnetic field in Fig. 2b–d.

**Figure 3:**
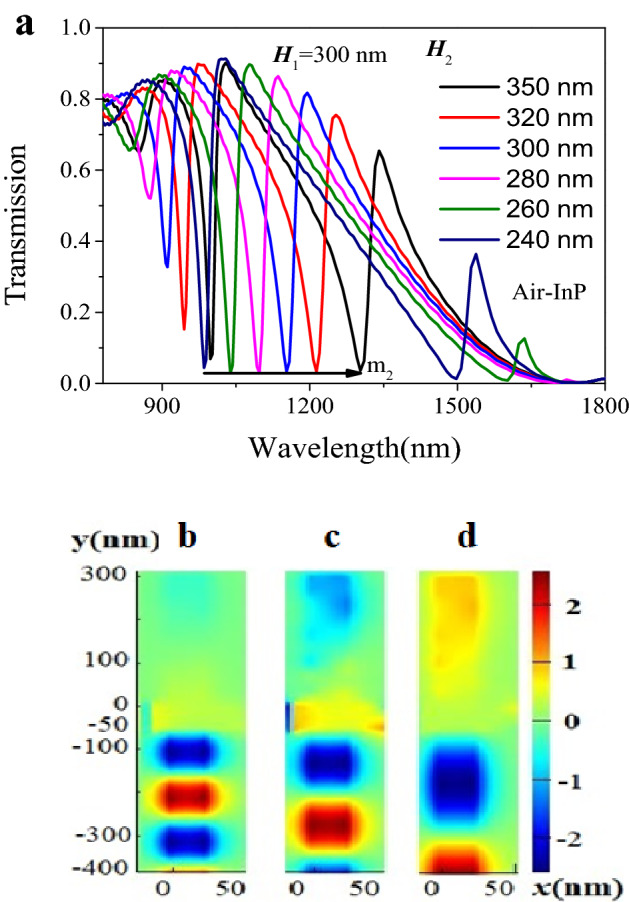
(**a**) The relationship between Fano resonance and the height *H*_2_ in the range from 780 to 1800 nm. (**b**–**d**) Magnetic field distribution of different modes at different resonance wavelengths 998 nm, 1302 nm and 2079 nm, respectively. Red and blue represent two different vibrations. The structural parameter was *H*_1_ = 300 nm.

When the zero order dark mode from the Air cavity and j-order bright modes (j = 1, 2 and 3) from the InP cavity were superimposed, some Fano resonance of the modes m_j_ (j = 1, 2 and 3) could be obtained in Fig. 3a, which showed the transmission spectra of different InP-Air structures. In the case of only air cavity, the transmission spectrum was very wide passband, which covered the resonance wavelengths of the modes m_j_ (j = 1, 2 and 3) and whose central wavelength was lied at about 1710 nm. For the mode m_1_, more obvious Fano resonance was observed when *H*_2_ = 240 nm and 260 nm. However, the most obvious Fano resonance came from the mode m_2_ whose resonance valley shifted to the long wavelength with the *H*_2_ increase. According to the main resonance peak^22–23^ of the mode m_2_ in the Fig. 3a, the quality factor (QF) and the extinction ratio (ER) could be calculated^19,20,23,26^. The quality factor Q was defined as the ratio of the resonance wavelength λ_0_ and the full width Δλ between the peak and the antipeak of the transmission (Q = λ_0/_Δλ). The extinction ratio (Ext) was ten times the logarithm of the ratio of the maximum transmission light intensity *P* to the minimum transmitted light intensity *P*_0,_ namely, Ext = 10lg(*P*/*P*_0_). A high-quality factor meant lower light energy loss in a resonant cavity. Moreover, the lager extinction ratio was implied the better quality of the resonator. For the different InP-Air structures with the length *H*_2_ from 240 to 350 nm (240 nm, 260 nm, 280 nm, 300 nm, 320 nm and 350 nm), the quality factor QFs calculated were about 44, 30, 29, 28, 34 and 37, respectively. The ERs obtained were around 13, 18, 15, 14, 31 and 12 dB, respectively. These results showed that the QFs and the ERs were a function of the height ***H***_**2**_. There was lower light energy loss in a resonant cavity and better quality of the resonator for the length ***H***_2_ = 240 nm and 320 nm. In other words, when the height ***H***_2_ was not higher than 240 nm and not less than 320 nm, the waveguide structure with InP-Air cavities had good filtering performance. In addition, it was obtained that the addition of air cavity did not change the resonant position which was dependent on structure sizes of the cavity InP. Take *H*_2_ = 350 nm as an example, for the three resonance valleys at 998 nm, 1302 nm and 2079 nm, it was observed the magnetic field distribution did not change before and after the addition of air cavity. Additionally, it was obtained that the magnetic field distribution of the zero-order resonance in the air cavity, as shown in Fig. 3b–d.

**Figure 4:**
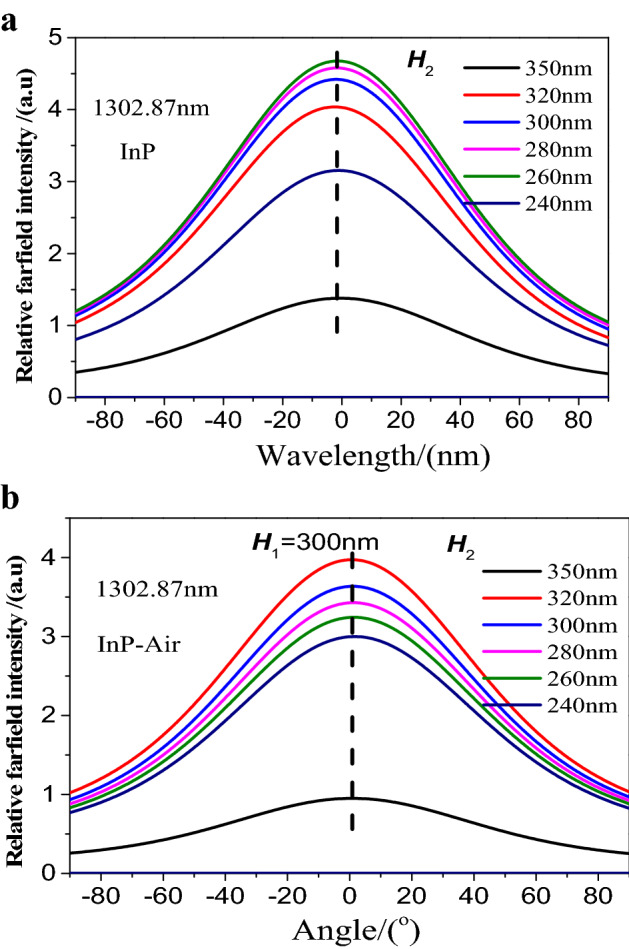
Farfield with vavious angle from − 90° to 90° and changeable height *H*_2_ from 240 to 350 nm before (**a**) and after (**b**) the addition of air cavity under 1302.87 nm monitoring.

Before the addition of air cavity, under 1302.87 nm monitoring, the relative farfield intensities *I* varied with the height ***H***_2_ from 240 to 350 nm and was a function of height ***H***_2_, namely, *I* = *I*(***H***_2_), as shown in Fig. 4a. The shape of the curve was Guassian Spot with periodic structure, whose symmetric center was located at about *θ* = − 2.2°, as shown in the black dash. The relative farfield intensity *I* gradually decreased with the height ***H***_2_ from 260 to 350 nm. The maximum relative intensity *I*(260) was about three times that of the minimum *I*(350), *I*(260) = 3*I*(350). After the addition of air cavity, it was observed that the relative farfield intensities *I* was also a function of height ***H***_2_, as shown in Fig. 4b. The symmetric center of the curve with periodic structure was located at about *θ* = 0°, as seen in the black dash. The relative farfield intensity *I* gradually increased with the height ***H***_2_ from 240 to 320 nm. The maximum relative intensity *I*(320) was about four times that of the minimum *I*(350), namely, *I*(320) = 4*I*(350). Therefore, the change of structural parameters could be obtained according to the change of far-field relative intensity^20^.

## Discussion

Influence of InP coupling cavity on Fano resonance of sub wavelength MIM waveguide was studied in this paper. Some novel results were obtained. For different InP structures without air cavity, it was observed the resonance peaks of mode m_j_ (j = 1, 2 and 3) redshifted with *H*_2_ increase and the resonant wavelengths of mode m_j_ (j = 1, 2 and 3) were a function of the height *H*_2_. For the different InP-Air structures with the length *H*_2_ from 240 to 350 nm, the resonance valley of the mode m_2_ shifted to the long wavelength. In addition, before and after the addition of air cavity, the relative farfield intensities *I* was a function of the height *H*_2_. Therefore, InP as discrete state of Fano resonance could be used as the filling dielectrics of Fano resonance in the MIM waveguide.

## Methods

### Numerical simulations

In the letter, the MIM plasmonic waveguide coupled with InP cavity was investigated using the finite-difference time-domain (FDTD, Lumerical Computational Solutions Incorporation) with a perfectly matched layer absorbing boundary condition. A plane wave with the electric field parallel to the *x* axis illuminates normally the periodic structure. The grid sizes in the *x* and *y* directions were 2 nm.
